# GuaRD: Guaranteed robustness of image retrieval system under data distortion turbulence

**DOI:** 10.1371/journal.pone.0288432

**Published:** 2023-09-28

**Authors:** Hyerin Chung, Nakyung Lee, Hansol Lee, Youngsun Cho, Jihwan Woo

**Affiliations:** AI Research, CJ OliveNetworks, Seoul, Korea; Mirpur University of Science and Technology, PAKISTAN

## Abstract

Image search systems could be endangered by adversarial attacks and data perturbations. The image retrieval system can be compromised either by distorting the query or hacking the ranking system. However, existing literature primarily discusses attack methods, whereas the research on countermeasures to defend against such adversarial attacks is rare. As a defense mechanism against the intrusions, quality assessment can complement existing image retrieval systems. “GuaRD” is proposed as an end-to-end framework that uses the quality metric as a weighted-regularization term. Proper utilization and balance of the two features could lead to reliable and robust ranking; the original image is assigned a higher rank while the distorted image is assigned a relatively lower rank. Meanwhile, the primary goal of the image retrieval system is to prioritize searching the relevant images. Therefore, the use of leveraged features should not compromise the accuracy of the system. To evaluate the generality of the framework, we conducted three experiments on two image quality assessment(IQA) benchmarks (Waterloo and PieAPP). For the first two tests, GuaRD achieved enhanced performance than the existing model: the mean reciprocal rank(mRR) value of the original image predictions increased by 61%, and the predictions for the distorted input query decreased by 18%. The third experiment was conducted to analyze the mean average precision (mAP) score of the system to verify the accuracy of the retrieval system. The results indicated little deviation in performance between the tested methods, and the score was not effected or slightly decreased by 0.9% after the GuaRD was applied. Therefore, GuaRD is a novel and robust framework that can act as a defense mechanism for data distortions.

## Introduction

The vast mass of data could easily become a mess rather than a gold mine. If a distortion is applied, searching a target image becomes difficult. Image retrieval techniques have improved over time. Conventional studies are based on pixel-level localized feature information and re-ranking, starting from bag-of-words in visual feature data [1] and pixel-level comparison methods such as the scale invariant feature transform (SIFT), speed up robust feature (SURF), and oriented fast and rotated brief (ORB) [2–4]. The results of these long-standing systems have been well explicated [5]. In the image retrieval domain, deep learning methods have been utilized to extract adequate features. Compared with conventional approaches, feature value extraction may be performed fast under the premise that the network is well trained. Not only global-level (context-level) features but also local-level features are available for capture [6–10]. Furthermore, utilization of multi-modal [11–14] and transformer models [15, 16] further increases the accuracy.

**Fig 1:**
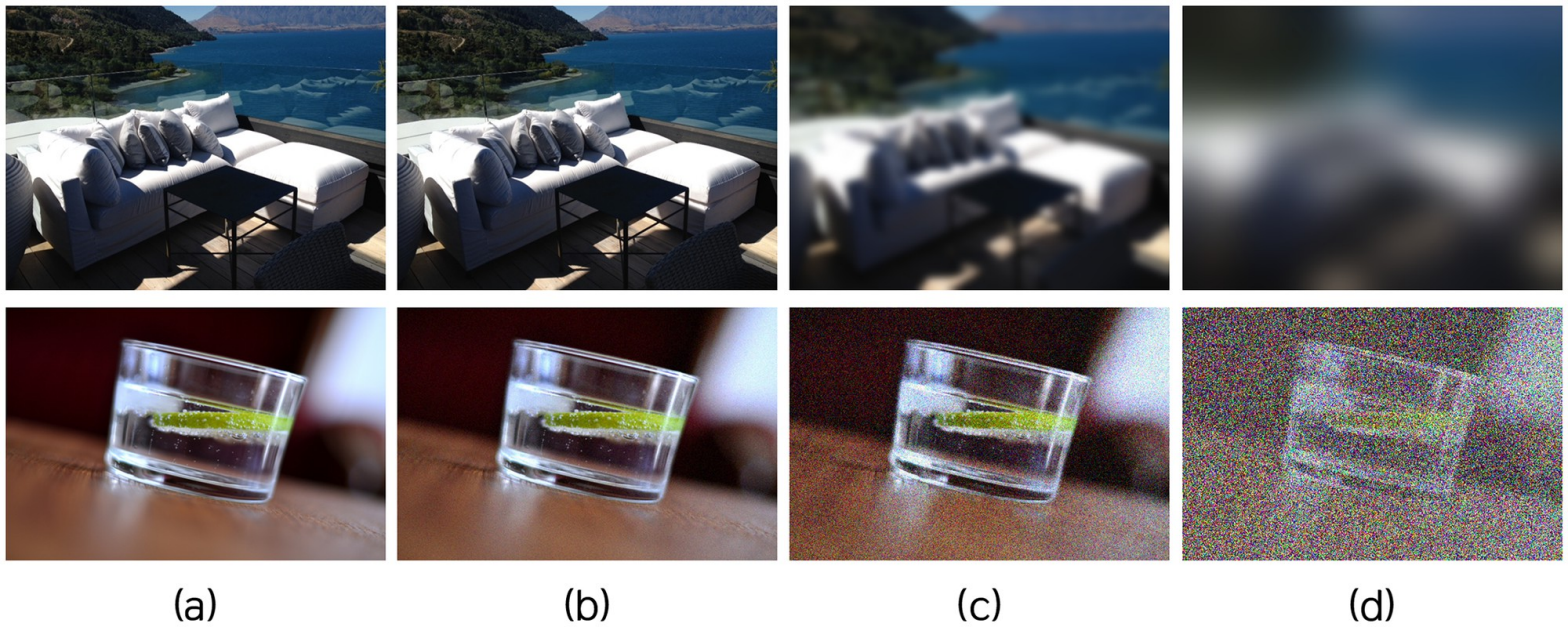
Examples of data distortion from the Waterloo dataset. (a) is a set of original images and (b), (c), and (d) are the sets of distorted images. The scale of distortion is set to 1, 3, 5 for each set, from the lowest to the highest.

Because the system makes judgments based on the extracted image vector values, the judgment becomes unreliable if the image vector itself is corrupted or if the differences between the vectors are not obvious, making searching difficult. In real-world scenario, data are unintentionally and constantly distorted by copying, transfer, download, and upload. The resolution of the image is altered and unwanted noise is inserted in the image. Fig 1 demonstrates an example of data piracy. An unauthorized downloaded image can be uploaded to another site that doesn’t have a defense with a slight variation. In addition, data are tampered with and mass-produced by generative models. Handling adversarial attacks and ensuring uniqueness is an important issue.

**Fig 2:**
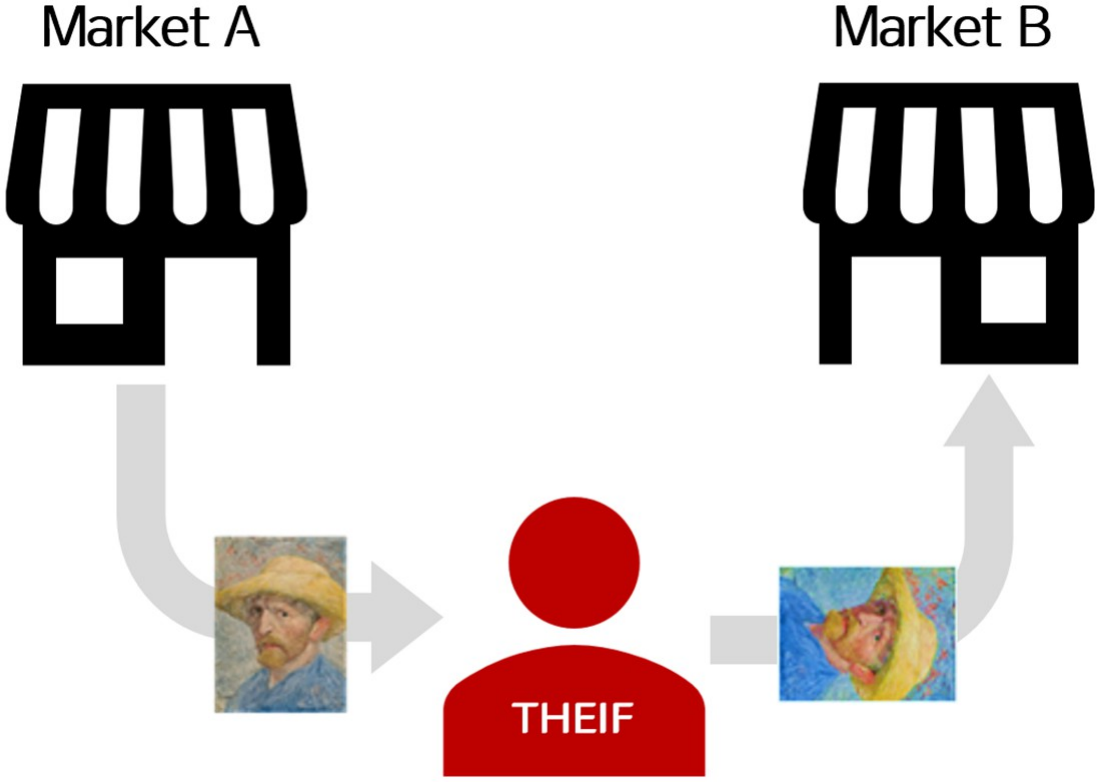
Example of vulnerability in image retrieval system. Without defense mechanisms, anyone can download data from one market (or website) and publish those to another. During the upload process, the data are mostly distributed, whether intended or unintended. In this scenario, a thief downloads the image from market A and adds noise that is not noticeable for humans. Because no restrictions exist in market B, the incoming image will be considered as a pristine version. The example artwork aligns with the figure in S1 Fig.

Numerous techniques have been introduced to attack image retrieval systems; developing methods to defend against such attacks is difficult. Defense techniques have two implications for image search. First, the system is able to clearly distinguish between original and distorted images for rankings and is able to restrict one or the other. Second, the system will be able to prevent images from being distorted. For domains that should be robust and reliable, the originality of data must be prevented. In non fungible token(NFT) or e-commerce market, for example, people could download the image and upload the distorted image in another market until the market realizes that it is a duplicated version. Fig 2 illustrates a possible scenario of the image retrieval systems without any restrictions.

In the next subsection, we briefly explained the research related to this study. Section 2 describes several benchmarks dataset and the structure of the model. To verify how well this model defends distorted images, experiments were conducted. Section 3 presents the results of the experiment from the perspective of precision and robustness. Section 4 illustrates the limitations of our approach and summarizes the contents of the thesis while concluding the paper. The contributions of this paper are as follows:

- We present a novel framework, “GuaRD”, to defend the retrieval system against adversarial attacks and data perturbations while easily leveraging the existing system. GuaRD is highly compatible with other engines.
- We present an approach that prevents adversarial attacks while searching relevant items without using additional technologies, such as block-chain.
- We applied the image quality assessment to the retrieval system as a regularization term for the first time.

### Related work

#### Image Retrieval(IR) and feature extraction

Recent deep-learning-based models have become state-of-the-art in connection with various tasks in computer vision and have great leverage in extracting significant visual representations from images in the image retrieval tasks. Specifically, convolutional neural network (CNN)-based methodologies are widely studied because traditional bag-of-word techniques have demonstrated improvements by utilizing feature vectors from the activation of well-known pre-trained CNNs as image descriptors.

R-MAC [17] proposed a method for extracting the representation of images by combining max-pooling at the back of the CNN architecture, which does not include a fully connected layer. This makes it possible to simultaneously obtain both global and local representations. However, it has the limitation of including information of unnecessary regions, such as the background, distant objects, and other trivial aspects when exploring local features within the image. To address this problem, Gordo et al. [18] presented a method for obtaining local features of meaningful object localization using region proposal networks (RPNs). Furthermore, they described a Siamese network that computes similarities between images while sharing the weights of convolutional layers by feeding the similar positive, non-similar negative, and query images, which was also constructed in the end-to-end deep learning architecture DIR [19]. Ahmad Faiyaz [20] introduced a deep image retrieval method that combines neural network interpolation and similarity-based indexing to enhance image retrieval. By integrating deep learning techniques, the proposed approach improves accuracy and effectiveness efficiently in retrieving images.

Approaches with transformer models combined with a retrieval module have embraced image retrieval in computation elapsed time, extracting localized (detail) information. Nath et al. [21] utilized a pre-trained big transfer (BiT) model with triplet loss. The BiT model was trained using a large, supervised dataset (tf_flower) to extract features and later fine-tuned for the target task. The extracted features were later used in searching for a similar target using a trained K-nearest neighbor (KNN) network for image retrieval. FIRe [22] proposed a local feature integration transformer (LIT), which has a transformer-like architecture that extracts super-features that are local features along with global features from image data. The ASMK model [7] was used to support the extraction in local-features, which is used from the beginning of the training phase instead of only at the retrieval phase. SSL-ViT-16 [23] showed that it is possible to extract meaning from unlabeled data by applying the feature embedding method with a self-supervised vision transformer in the zero-shot image retrieval task. Revaud et al. [24] proposed a new method to optimize the global mean average precision (mAP) for image retrieval by leveraging recent advances in listwise loss formulations. They proposed a new method and benchmark by simultaneously considering multiple images at each iteration and eliminating the need for ad-hoc tricks.

As the components that make up the query required for image retrieval are becoming more diverse, many recent works have studied how to combine multi-modal queries. Gordo et el. and PCME [11, 25] used combination of visual and textual embedding based on similarities. ARTEMIS [13] proposed to use two attention mechanisms, one executing a target image and reference image with text as input, and the other computing a score for how well the target image matches the text. CAISE [14] introduced a conversational agent that can not only edit the visual properties of images but also retrieve images from user requests. It can embed images and utterances via faster RCNN / positional encoding and the LSTM architecture and then generates an executable command for editing or retrieving images via attention mechanisms that can compute the similarity of each of the two embedding vectors. Considering the improvement by adopting additional data, multi-modal is surely a considerable domain.

Feature extraction, an important underlying technology in the field of image retrieval, is utilized in various fields such as object recognition, detection, and medical analysis. Damaneh et al. [26] presented a method for recognizing static hand gestures in sign language. It utilizes a CNN along with feature extraction techniques using the ORB descriptor and Gabor filter for accurate hand-gesture recognition. Yang et al. [27] examined integrating deep learning techniques into face perception technology to improve accuracy in diverse lighting conditions. By leveraging advanced algorithms, the proposed approach enhances face recognition in varying illumination intensities. Our strategy closely aligns with the approach of leveraging advanced deep learning algorithms. Qureshi et al. [28] focused on a method called radiogenomic classification, which uses a combination of different types of data (multi-omics fused feature space) to determine the MGMT promoter methylation status. Their goal was to develop a non-invasive diagnostic approach using mpMRI scans that requires minimal invasiveness.

#### Image Quality Assessment (IQA)

The quality evaluation of the image could be classified as; full-reference(FR) and no-reference based on whether the reference image(clean, pristine image) is required. PSNR and SSIM [29] approaches are two popular traditional full-reference image quality assessment(FR-IQA). PSNR is a pixel-based metric that measures the difference between two images in pixel-level values. SSIM measures the structural similarity considering the luminance, contrast, and structural information of the images. DeepSim, LPIPS, and IQT apply deep learning as an approach [30–32]. Each combined VGGNet, transformer, and attention to improve the accuracy.

No-reference image quality assessment(NF-IQA) methods evaluate the image without having access to a pristine image. Natural image quality evaluator (NIQE), BRISQUE [33, 34] are traditional no-reference approaches. NIQE is a statistical approach that captures and learns features of pristine images, such as texture and contrast, and predicts the image quality. BRISQUE uses a support vector regression (SVR) model to predict the image quality. Deep learning-based approaches have also been proposed for NF-IQA [35–42]. Multi-scale architecture [38, 39], transformer model [38, 40, 41], and contrastive learning [42, 43] have recently been introduced.

#### Adversarial attacks

Studies have been conducted on the security of the ranking system. In particular, in the case of images, various definitions of distortion exist. The general approach for an adversarial attack is distortion by adding noise to the image in the query stage [44–47].

ZQBA [44] has beenintroduced a zero-query attack method for attacking content-based image retrieval(CBIR) systems in a black-box setting where no knowledge about the system is available. The method is based on an ensemble of models that perform optimization using some surrogate feature-extraction models and complement the optimized results. QAIR [45] performs a query-based attack against the image retrieval system under black-box settings. It has a relevance-based objective function for quantifying the attack effects and a recursive model stealing method to improve the query-attack efficiency. The system is capable of fooling commercial image retrieval systems such as Bing Visual Search with a few queries. NAG [46] is a method to achieve the most challenging black-box attacks in deep hash-based image retrieval. The relations between adversarial subspace and black-box transferability have been explored by using random noise as a substitute. The proposed model is an algorithm to estimate the adversarial region by introducing random noise, which is used to assess the capacity of different attacks. In PIRE [47], data perturbation is defined as a change that could not be easily caught by the human eyes but altered to disrupt the content-based retrieval system. It analyzes of adversarial queries in unsupervised methods, focusing on neural, local, and global features.

Additional approaches include attacking the ranking system to return the unexpected results as a prediction [48, 49]. UAP [48] includes a set of universal attack methods against image retrieval systems to cause the system to return unrelated images as top results in the ranking list. The authors generated universal adversarial perturbations (UAPs) that can be applied to all query images using gradient-based optimization algorithms. This attack can significantly reduce the accuracy of the system by disrupting the neighborhood relationships between features in the images used for retrieval. DAIR [49] is an efficient query-based attack method for image search systems, using projected natural evolution strategies (PNES) to generate adversarial perturbations that flip the top-K search results. PNES is an optimization algorithm that uses natural evolution strategies to search for the optimal perturbation vector, incorporating new projection operators, utility functions, and optimization techniques to ensure that the perturbed images remain visually similar to the original images.

## Materials and methods

### Dataset

For the realistic data corruption, we conducted experiments on two image quality assessment benchmarks. In addition to the addition of noise, we experimented with Waterloo Benchmark [50] and PieApp Benchmark [51] in terms of large data volumes and different types of distortions that can actually occur, such as data loss due to compression.

#### Waterloo exploration database

Waterloo Exploration Database contains 4744 original and 94880 distorted images. It is proposed to compensate for the limited content variations of previous benchmarks. The original image is distorted into four distortion types and five distortion levels.

#### PieAPP

PieAPP benchmark is utilized for training image-error prediction algorithms by providing reference images and their distorted versions labeled with probability of preference. It uses a subset of 200 reference images from the Waterloo dataset, and 40 human subjects were queried to ensure reliable probability labels. Total 19,680 distorted images were generated, and we captured distortion in aspects of common image artifacts (e.g., additive Gaussian and speckle noise), HVS (e.g., non-eccentricity, contrast and sensitivity); and complex algorithms (e.g., deblurring, denoising, super-resolution, and compression).

### Methods

**Fig 3:**
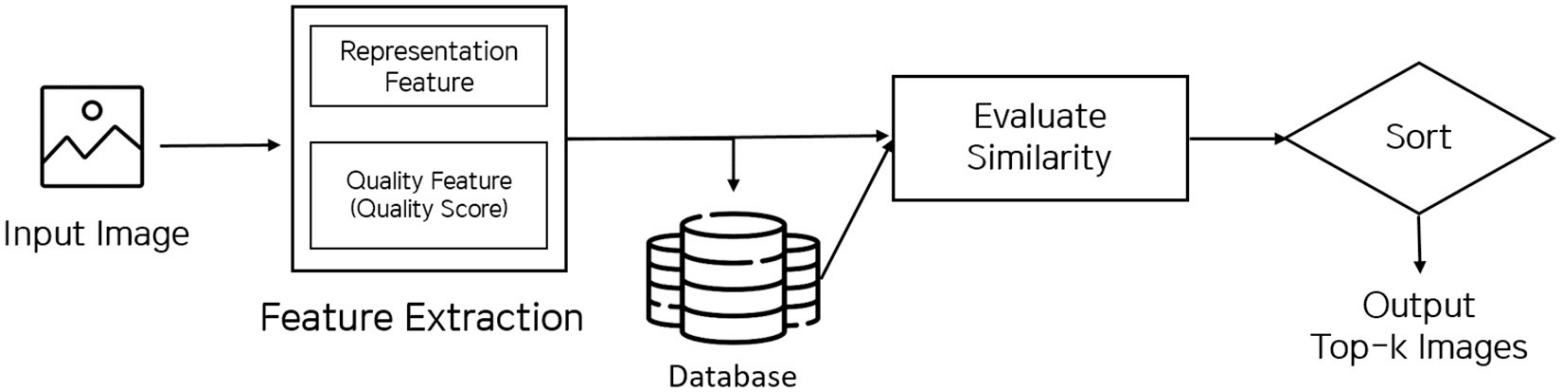
Abstract description of the retrieval system by data processing flow. When an image datum is input, it will be compressed by the feature extraction model as an embedded vector. The extracted vector will be directed to the database if it is not previously stored. Moreover, it is directed to the evaluation model to compare with candidate data and rank the items.

GuaRD is a two-stage frame work: 1) feature extraction and 2) ranking system. Fig 3 shows the overall structure of the system according to the process flow of analyzing a given image. Once the image is input as a query, its feature will be extracted by the module and will be converted to a vector value. The extracted image vector will be stored with other metadata of the images. By scanning the database and calculating the similarities, the system recommends possible candidates.

#### Feature extraction

In this stage, two features should be collected by the module: representational and quality. in the aspect of the representational feature, it contains information such as shape and color. this information has already been utilized in pre-existing image retrieval systems; therefore, it will also be utilized in this framework. The quality information is obtained by the quality assessment models. To ensure that the original image is ranked at the top and the distorted image is ranked lower than the original, two features are normalized and balanced.

#### Representation feature

Feature extraction methods vary depending on the purpose of the task, such as classification or detection. For simple CBIR system, we chose the self-supervised learning models for the feature extraction. However, the extraction module of GuaRD is highly compatible with other methods and could be replaced by other models as required.

The BYOL model is learned by leveraging the idea of “positive pairs” consisting of an original image and its augmented version. We expected a model that could distinguish it from other data while focusing on the features of each data itself, even if similar data existed. Utilizing self-supervised methodologies, several models were evaluated on a library framework [52]. Table 1 presents the prediction accuracy of each model measured by mean precision, as indicated in the form of ‘mp@k’. The values were edited to compare performance against BYOL, set to 1, and correspondingly compared with the others. It is a preliminary predictor that accurately shows results. Regarding the top-1 prediction, the SupCon model was twice as accurate as the other models. In the top-3 and top-5 predictions, when compared with other models, the accuracy level was inferior. BYOL model, by contrast, showed stable predictive performance. Hence, the final feature extraction model was designed based on the BYOL model.

Because the original purpose of the system was to focus on simple similarity comparison rather than classification, the used data also do not have separate label values. As an adequate methodology for the feature extraction module, BYOL, one of the self-supervised learning models, was utilized.

**Table 1:** Mean precision at rank K (mP@K) with pre-trained models.

	mp@1	mp@3	mp@5
**BYOL (Baseline)**	**1**	**1**	**1**
SupCon	2	0.533	0.507
SimCLR	1	0.955	0.926
SimSiam	1	0.893	0.867
NNCLR	1	0.88	0.878
ReSSL	1	0.828	0.816
MoCo-v2+	1	0.823	0.798

Seven models were tested for precision throughout multiple self-supervised contrastive learning methods. BYOL’s result was used as a baseline and set to 1 for comparison. Additionally, the results of other models were modified based on the baseline.

**Fig 4:**
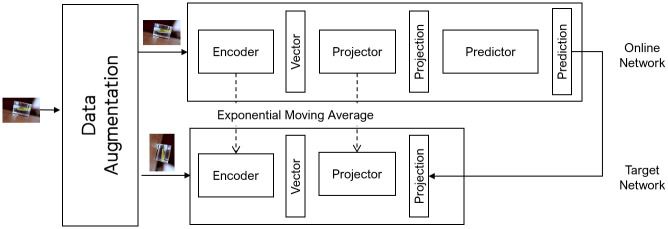
Proposed feature extraction network based on the BYOL structure. The backbone of the feature extraction network is based on the BYOL network. Through the network, a given image is converted into an embedded vector, which is a vector learned by the projector of the model.

Fig 4 and the following equation are based on the schematics of the existing study on BYOL [53]. It consists of two networks (online and target) and passes the same image through different data augmentation steps. The network learns the essence feature by predicting the projection result $z_{\in }^{\prime }$ of the target network with *q*_*θ*_ and (*z*_*θ*_). Each (*θ*, *γ*) describes trained weights and the exponential moving average. To prevent model collapse, the target network updates the values of the online network using the exponential moving average method [53]. Eq 1 obtains the loss value and normalizes the batch. L_*θ*,*γ*_ is a loss function optimized by minimizing the difference between the projection of *γ* and prediction result of q_*θ*_(*z*_*θ*_). Because the network is symmetrical, the loss function of the other half network can be expressed as ${\tilde{L}}_{\theta ,\gamma }$.

The loss function of BYOL presented in Eq 2 is optimized for the online network. *η* denotes the learning rate. In training, the model is intended to learn through the stochastic optimization step with the total set of loss functions. The parameter values of the target network are updated in Eq 3. The weighted target value is aggregated with the updated value *θ* from the online network.

$$\begin{array}{c} L_{\theta ,\gamma }≜∥{\bar{q}}_{{}_{\theta }}\left(z_{\theta }\right)-{\bar{z^{\prime }}}_{\gamma }{∥}_{2}^{2}=2-\frac{2*<q_{\theta }\left(z_{\theta }\right),z_{\gamma }^{\prime }>}{∥q_{\theta }\left(z_{\theta }\right){∥}_{2}*∥z_{\gamma }^{\prime }{∥}_{2}} \end{array}$$ (1)

$$\begin{array}{c} \theta =optimizer(\theta ,\nabla_{\theta }\left(L_{\theta ,\gamma }+{\tilde{L}}_{\theta ,\gamma }\right),\eta ) \end{array}$$ (2)

$$\begin{array}{c} \gamma =\tau *\gamma +(1-\tau )\theta \end{array}$$ (3)

#### Quality feature and integration

To obtain the quality information of the image, CONTRIQUE, which is no-reference image quality assessment model is adapted. The feature extracted from CONTRIQUE contains information on the scale and types of distortion which are applied in the model. The distances calculated from two aspects (representation and quality) are balanced and merged to execute the similarity of the images. In other words, the quality of the image is used as a regularization term. Eq 4 is the equation that calculates the integrated distance. Determining the appropriate *α* value depends on the specific characteristics and configuration of a given dataset. For this experiment, we used an the value of 0.9.

$$\begin{array}{cc} & d_{\text{aggregated}}=\alpha *d_{representation}\left(x\right)+\left(1-\alpha \right)*d_{quality}\left(x\right) \end{array}$$ (4)

### Ranking

To estimate the similarity between images, integrated feature vectors are used and judged based on the distance between vectors. If the distance is closer, the images are more similar. To search the n number of the most similar items, the nearest neighbor algorithm is used. An open source library, faiss [54], is applied, which is optimized for the GPU. The distance is calculated based on the l2-norm distance metric.

## Results

To assess the performance of the framework, experiments were conducted from two perspectives: precision and robustness. An NVIDIA GPU (GeForce RTX 2080 Ti Rev, GeForce RTX 3090) on Ubuntu 20.04.3 was used for model learning.

An additional IR model proposed by Revaud et al. [24] was applied in addition to BYOL to test the robustness and generality of the framework(the model will be notated as ‘dirtorch’ throughout the experiment). Among multiple studies, dirtorch exhibited similar performance to BYOL model with TID2013 [55]. Each original model is counted as the baseline, and notation of GuaRD applied version of the x model is depicted as GuaRD(x). Moreover, the IQA model (CONTRIQUE) was tested to compare the synergy with IR models. Three tests were conducted for each benchmark. First, the original(pristine) image was assumed to appear on the higher rank of the prediction. Second, the query image was assumed to be ranked lower than the baseline because the balanced feature would ensure the distorted images to go under. However, we still want to emphasize that the top-K result of prediction must contain both the original data and distorted input query. Therefore, the retrieval system should predict relevant items at the top of the prediction.

### Metric

Mean reciprocal rank (mRR) quantifies the quality of the ranking by calculating the average reciprocal of the first relevant item ranking, and it indicates how well the system retrieves the most relevant results. For the first and second assumptions, mRR is used to ensure that the target properly is ranked. If the item is ranked in the top-5, the value of mRR is 0.2 (1/5). The closer the value to 1, the higher the target will be ranked.

Mean average precision (mAP) measures the average accuracy for different levels of recall to comprehensively assess the accuracy and completeness of the prediction. It is used as an accuracy metric for the last statement to make sure that the relevant items (distorted images generated from the same original image) are predicted. From scale 0 to 1, a higher mAP value indicates that the more relevant items were predicted.

Across all evaluations, the model’s performance is the most pronounced with middle image distortion (scale-3 for Waterloo and scale-4 for PieAPP). This indicates effective leveraging between the quality and representation works in the middle scale of image distortion.

### Waterloo benchmark result

Because the scale of distortion in the Waterloo benchmark is applied from 1 to 5 (easy to hard) based on the given benchmark, each result was analyzed based on the scale. Overall, GuaRD-applied retrieval systems exhibited more robust and reliable performance compared with the bare model. Table 2 presents the mRR result of the original image, which depicts where the image is ranked. The GuaRD-applied retrieval system (GuaRD(dirtorch) and GuaRD(BYOL)) ranked the highest, stating that the original image is ranked around top-4 or top-5, whereas it is ranked around top-6–8 in the native retrieval systems. Considering the result of CONTRIQUE, it appears that the feature vector extracted solely by IQA models is hard to be used for retrieval systems. A bigger the scale number indicates more data perturbation applied to the data and so does the aspect of mRR value is lower.

**Table 2:** mRR result of searching the original image.

scale	CONTRIQUE	dirtorch	BYOL	GuaRD(dirtorch)	GuaRD(BYOL)
1	0.000	0.075	0.165	**0.323**	**0.218**
2	0.000	0.036	0.166	**0.276**	**0.219**
3	0.001	0.047	0.166	**0.127**	**0.219**
4	0.176	0.177	0.166	**0.171**	**0.220**
5	0.005	0.224	0.166	**0.372**	**0.220**
Avg	0.036	0.112	0.166	**0.254**	**0.219**

Table 3 presents the mRR result of searching the input image. The GuaRD appiled models marked lower value, which indicates that the query image was ranked lower in the prediction of GuaRD compared with the bare system. However, BYOL demonstrated reliable results. The overall performance merely decreased by the scale of distortion. GuaRD-applied dirtorch’s results changed dramatically; it reduce to 0.612. This implies that the query image is ranked at least second or third. The results demonstrate that GuaRD’s prediction can find both the original and the distorted input image at the top of the list.

**Table 3:** mRR result of searching the query image.

scale	CONTRIQUE	dirtorch	BYOL	GuaRD(dirtorch)	GuaRD(BYOL)
1	0.965	0.964	0.951	**0.391**	**0.946**
2	0.965	0.965	0.952	**0.536**	**0.948**
3	0.964	0.964	0.952	**0.857**	**0.948**
4	0.903	0.903	0.951	**0.682**	**0.945**
5	0.969	0.969	0.950	**0.595**	**0.946**
Avg	0.953	0.953	0.951	**0.612**	**0.947**

Table 4 shows the results of relevant item prediction. We determined that the more items that have the same source as the query item are predicted higher, the better is the prediction. Difference in scale does not appear to effect the bare models (CONTRIQUE, dirtorch, and BYOL), and results of retrieval systems or GuaRD-applied versions are broadly similar. However, the GuaRD-applied version performs slightly worse in scale 3 and 4. At scale 3, the quality vector appears to be more influential in the prediction results, as the distortion in the image is more noticeable to the human eye. The prediction by the IQA model merely ranks the relevant items. Considering that the goal of CONTRIQUE is to analyze the quality features and not the visibility, independently using its feature is not suitable.

**Table 4:** mAP result of relevant item prediction.

scale	CONTRIQUE	dirtorch	BYOL	GuaRD(dirtorch)	GuaRD(BYOL)
1	0.119	0.232	0.211	**0.267**	**0.245**
2	0.120	0.231	0.211	**0.253**	**0.231**
3	0.118	0.232	0.212	**0.175**	**0.175**
4	0.115	0.232	0.212	**0.152**	**0.129**
5	0.125	0.232	0.212	**0.268**	**0.248**
Avg	0.119	0.232	0.212	**0.223**	**0.206**

### PieAPP benchmark result

Similar to the previous experiment, the applied distortion varied in the PieAPP benchmark’s distortion; therefore it was separately analyzed by scale. Overall, GuaRD-applied systems were more stable than the native models.

Table 5 shows the mRR result of the unspoiled image. The improvement between models appears minor. Nevertheless, on average, GuaRD-applied retrieval systems find the original image better than the bare system.

**Table 5:** mRR result of searching the original image.

scale	CONTRIQUE	dirtorch	BYOL	GuaRD(dirtorch)	GuaRD(BYOL)
1	0.030	0.079	0.088	**0.127**	**0.125**
2	0.035	0.071	0.097	**0.116**	**0.139**
3	0.048	0.096	0.133	**0.149**	**0.172**
4	0.078	0.154	0.211	**0.221**	**0.238**
5	0.036	0.108	0.172	**0.179**	**0.203**
6	0.022	0.104	0.172	**0.183**	**0.209**
7	0.020	0.097	0.168	**0.184**	**0.208**
Avg	0.038	0.101	0.149	**0.166**	**0.185**

Table 6 illustrates the mRR result of searching the input image from the system. The performance gap is dramatic between the GuaRD-applied version and the unsupported version. The mRR value of GuaRD-applied models was significantly lower by approximately 0.2, which indicates that the support of the GuaRD system drags the distorted images lower regardless of whether it is an exact item or not.

**Table 6:** mRR result of searching the query image.

scale	CONTRIQUE	dirtorch	BYOL	GuaRD(dirtorch)	GuaRD(BYOL)
1	0.978	0.976	0.978	**0.841**	**0.854**
2	0.981	0.980	0.980	**0.802**	**0.880**
3	0.988	0.985	0.986	**0.758**	**0.893**
4	0.981	0.981	0.981	**0.714**	**0.900**
5	0.989	0.989	0.988	**0.703**	**0.851**
6	0.986	0.992	0.988	**0.654**	**0.835**
7	0.983	0.981	0.988	**0.624**	**0.830**
Avg	0.984	0.983	0.984	**0.728**	**0.863**

The accuracy result for predicting the relevant items in PieAPP benchmark is consistent with that of the previous test. All the retrieval system models display comparable results, as shown in Table 7. The reduced effect of the application of GuaRD is minimal. CONTRIQUE’s prediction result is slightly inadequate compared with others by 0.05.

**Table 7:** mAP result of relevant item prediction.

scale	CONTRIQUE	dirtorch	BYOL	GuaRD(dirtorch)	GuaRD(BYOL)
1	0.224	0.291	0.291	**0.291**	**0.275**
2	0.241	0.293	0.292	**0.293**	**0.287**
3	0.248	0.293	0.293	**0.293**	**0.291**
4	0.255	0.293	0.293	**0.293**	**0.293**
5	0.217	0.292	0.292	**0.292**	**0.290**
6	0.226	0.293	0.292	**0.293**	**0.290**
7	0.224	0.293	0.293	**0.293**	**0.291**
Avg	0.233	0.293	0.292	**0.293**	**0.288**

## Discussion

In this paper, we emphasized the need for defensive techniques and impact of an adversarial attack on image retrieval systems. As mentioned earlier, in industries utilizing the image retrieval system, such as search engines, NFT market platforms, and the e-commerce sector, the importance of prioritizing security cannot be overstated. Because data can be easily duplicated and compromises can go unnoticed, ensuring the system’s reliability becomes a challenging task. Hence, carefully considering to security measures in these domains is essential for protection against potential threats.

Previous studies are mostly focused on how to crack the system, and only a few represent a solution for both attack and defense aspects. GuaRD is a state of the art approach to defend systems against general distortions. However, it may have some possible limitations. Not all types of attacks were considered. Our framework is targeted for perturbation of data that are hard for human to recognize. Distortions such as data transformation or a direct attack for ranking stages should be prevented as well.

Because the architecture is a stack of multiple models, the execution time is higher than those of the existing image retrieval systems. A single query for top-10 item prediction consumes 0.054s, whereas with GuaRD more than one second is required. This is because the summation and sorting calculation is added during the ranking stage. The query time for retrieval system is an important aspect in the real world; however, we did not focus on the time efficiency in this study. Therefore, it needs to be optimized in the future.

### Conclusions

An image retrieval system could be vulnerable to adversarial attacks and data perturbation. As a defense mechanism against data perturbation, quality assessment could compensate the existing image retrieval system. GuaRD was suggested as an end-to-end framework that is highly compatible with other engines. The previously used retrieval system does not need replacement.

GuaRD uses the image quality as a regularization term. By ranking the original image higher and the distorted image lower, the system could prohibit the registration of duplicated or distorted versions of the image and assure robustness. To test the general usage of the framework, three experiments were conducted on both the IQA benchmarks(Waterloo, PieAPP) with multiple common data distortions applied. The mRR value of original image prediction has increased by 61% and distorted input query decreased by approximately 18% each. The result indicates that the support of the GuaRD framework on existing models appears to be effective. The accuracy of the third test was measured using mAP, and the performance merely decreased by 0.9%, which indicates that the relevant items are still ranked on the prediction. In summary, the GuaRD method demonstrated improved robustness in handling a wide range of distorted images, enhancing the performance of the existing image retrieval systems.

## Supporting information

S1 Fig Self-Portrait with Straw Hat. “Self-Portrait with Straw Hat”, 1887 by Vincent Van Gogh currently shown at the Detroit Institute of Arts.
(TIF)
