# Supplementary figures and images for: GuaRD: Guaranteed robustness of image retrieval system under data distortion turbulence
Source: PLoS One. 2023 Sep 28;18(9):e0288432. doi: 10.1371/journal.pone.0288432 (PMC10538669; doi:10.1371/journal.pone.0288432)

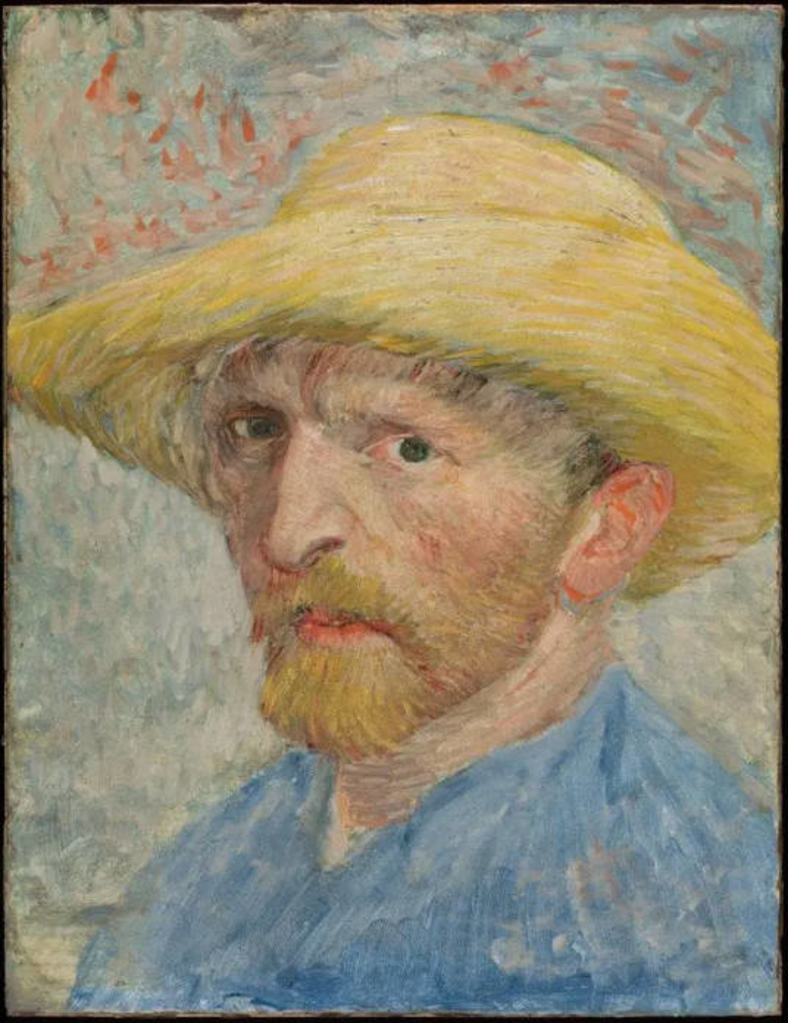

Supplement: S1 Fig — “Self-Portrait with Straw Hat”, 1887 by Vincent Van Gogh currently shown at the Detroit Institute of Arts. (TIF) [file pone.0288432.s001.tif]
